# Diamine Oxidase as a Therapeutic Enzyme: Study of Germination from Vegetal Sources and Investigation of the Presence of β-N-Oxalyl-L-α,β-diaminopropionic Acid (β-ODAP) Using LC-MS/MS

**DOI:** 10.3390/ijms24054625

**Published:** 2023-02-27

**Authors:** Rym Boulfekhar, Leanne Ohlund, Kathrina Mae Kumaresan, Meriem Megoura, Thomas D. Warkentin, Pompilia Ispas-Szabo, Lekha Sleno, Mircea Alexandru Mateescu

**Affiliations:** 1 Department of Chemistry & Center CERMO-FC, Université du Québec à Montreal, CP 8888, Branch A, Montreal, QC H3C 3P8, Canada; 2 Department of Chemistry & Research Chair Allerdys—Prevention of Allergies and Enteric Dysfunctions, Université du Québec à Montreal, CP 8888, Branch A, Montreal, QC H3C 3P8, Canada; 3 Crop Development Centre, Department of Plant Sciences, University of Saskatchewan, 51 Campus Dr., Saskatoon, SK S7N 5A8, Canada

**Keywords:** vegetal diamine oxidase, histaminosis, histamine, *L. sativus*, *P. sativum*, β-ODAP, LC-MS

## Abstract

Vegetal diamine oxidase (vDAO), an enzyme proposed to relieve symptoms of histaminosis, shows better reactivity with histamine and aliphatic diamines, as well as higher enzymatic activity than DAO of animal origin. The objective of this study was to evaluate the enzyme activity of vDAO from germinating grains from *Lathyrus sativus* (grass pea) and *Pisum sativum* (pea), and to verify the presence of a neurotoxin, β-N-Oxalyl-L-α,β-diaminopropionic acid (β-ODAP), in the crude extract obtained from their seedlings. A targeted liquid chromatography–multiple-reaction monitoring mass spectrometry method was developed and used to quantify β-ODAP in the analysed extracts. An optimized sample preparation procedure, involving protein precipitation with acetonitrile followed by mixed-anion exchange solid-phase extraction, allowed for high sensitivity and good peak shape for β-ODAP detection. The *Lathyrus sativus* extract exhibited the highest vDAO enzyme activity of the extracts, followed by the extract from pea cultivar Amarillo from the Crop Development Centre (CDC). The results have also shown that even though β-ODAP was present in the crude extract from *L. sativus*, its content was far below the toxicity threshold (300 mg of β-ODAP/kg body/day). CDC Amarillo showed 5000-fold less β-ODAP than the undialysed *L. sativus* extract. It was concluded that both species can be considered as convenient sources of vDAO for potential therapeutic use.

## 1. Introduction

Histamine intolerance (HIT), also referred to as enteral histaminosis, is a disorder caused by an excessive increase in histamine [1]. Histamine is a biogenic amine that plays an important role in the body as a neuromediator and immune modulator [2]. In contrast, HIT is defined by the impaired gastrointestinal breakdown of histamine from ingesting foods containing high levels of it [3], such as chocolate, certain cheeses, sauerkraut, tomatoes, and some red wines [4]. Among the causes of HIT is a reduced ability to metabolize histamine via the intestinal enzyme diamine oxidase (DAO) [5,6,7].

Considering that DAO is the main exogenic histamine-degrading enzyme with predominant activity in the gut, histamine excess in the gut lumen may trigger diarrhoea, abdominal pain, or constipation by enhancing neurosecretory functions and muscle contractility [8]. In addition, histamine excess may be linked to serious inflammatory bowel diseases, such as Crohn’s disease and ulcerative colitis [9]. DAO copper-containing amine oxidase (EC 1.4.3.22) is a homodimer and ubiquitous enzyme with a molecular mass in the range of 140–200 kDa. DAO catalyses the oxidative deamination of the primary amino group of histamine to imidazole acetaldehyde, consuming dioxygen with the simultaneous release of stoichiometric amounts of ammonia and hydrogen peroxide [10], as shown below.

Histamine + O_2_ + H_2_O → Imidazole-4-acetaldehyde + H_2_O_2_ + NH_3_

A deficiency in DAO activity can lead to the development of histamine intolerance, a clinical condition that can be treated with a low-histamine diet and oral DAO supplementation to improve intestinal histamine breakdown [11]. Pig kidney and some legumes seedlings are suitable sources for the formulation of a DAO supplement. In contrast to DAO of animal origin, vegetal DAO (vDAO) is believed to have higher specific activity, as well as being more acceptable and safer in terms of side effects [12]. Therefore, vDAO has been suggested as an effective therapeutic enzyme for oral administration to treat food histaminosis, allergies, and histamine-related enteric dysfunctions [13].

vDAO is found in different tissues (seeds, shoots, roots, seedlings, embryo axes, cotyledons, apexes, leaves) of a variety of plant species, including *Cicer arietinum* (seedlings), *Glycine max* (seedlings), *Lathyrus cicera*, and *Lathyrus sativus* (seedlings), *Pisum sativum* (seedlings, cotyledons, and embryos), *Vicia faba* (seedlings and leaves), and *Zea mays* (seedlings) [14]. vDAO is extracted from seedlings that are obtained after a period of germination. The germination process includes a set of physiological events that begins with the absorption of water by the dry seed and ends with extension of the embryonic axis and the emergence of hypocotyls and radicles [15]. At the cellular level, it is characterized primarily by the revival of respiratory activity by reactivating glycolysis, the Krebs cycle, and the respiratory chain [16]. Then, the reserves are mobilized by secreting hydrolytic enzymes followed by depolymerization and transport of reserves towards the embryonic cells in development, and finally, the activity of parietal hydrolases is increased, which reduces the mechanical resistance exerted by the tissues surrounding the embryo [17,18]. Adequate environmental conditions are necessary to ensure the proper development of seedlings. To obtain the most active DAO possible, the germination conditions, including temperature, water, and light, must be optimized to enhance growth of seedlings throughout germination [19,20,21,22,23].

Large concentrations of vDAO are mainly found in the etiolated seedlings of legumes of *Pisum sativum* (pea) and *Lathyrus sativus* (grass pea), where the total protein content can be as high as 4% [22,24]. Pea and grass pea seeds have a high nutritional value, with a protein content of 22–37% [25]. Grass pea is an affordable and wholesome food option in areas of North India and Africa that are vulnerable to drought and hunger [26,27,28]. However, due to the presence of β-N-oxalyl-L-α,β-diaminopropionic acid (β-ODAP), a free non-proteinogenic amino acid with neurotoxic effects, extensive production and consumption of grass pea seeds in food and feed have been limited. β-ODAP may generate an irreversible paralysis of the lower limb muscles in humans, resulting in “neurolathyrism” [27,29]. This chronic non-progressive motor neurodegeneration may be brought on by excessive ingestion of grass pea (300 to 400 g/day for 2 to 4 months) [30].

The main objective of this study was to explore the activity of vDAO in different cultivars of *P. sativum* developed in Canada and in *L. sativus* and determine their safety as a source of vDAO, in the context of potential toxicity from residual β-ODAP content. For this work, a targeted liquid chromatography–multiple-reaction monitoring (LC-MRM) tandem mass spectrometry assay was developed for the sensitive detection and quantitation of β-ODAP content in vDAO extracts.

## 2. Results

### 2.1. vDAO Activity in Different Seed Varieties

This study is an important part of a more extensive project focused on orally administered vDAO as a promising and effective therapeutic approach to alleviate histamine-related dysfunctions, particularly enteric ones. Various cultivars of *Pisum sativum* (*P. sativum*) have been compared with *Lathyrus sativus* (*L. sativus*) as sources of vDAO. One of the main parameters used to evaluate the obtained sources was the vDAO enzyme activity measured as the rate of oxidation of biogenic amines, such as putrescine or histamine, via the amount of hydrogen peroxide generated as a product of a reaction for extracts from different vegetal sources (Table 1), organs and tissues (Table 2), and germination time (Table 3).

Crude extracts from several Canadian field pea seed varieties obtained through the Crop Development Centre (CDC), as well as from *L. sativus* and yellow pea, were prepared to investigate their vDAO enzyme activities. vDAO enzyme activity, total activity, and specific activity of *L. sativus* (2.21 U/mL, 99.45 U, and 1.41 U/mg, respectively) were the highest compared with the other seed varieties of seeds (Table 1). When compared with other pea varieties, CDC Amarillo had the highest enzyme total activity and specific vDAO activity at (1.31 U/mL, 58.95 U, and 0.77 U/mg), respectively.

**Table 1:** DAO activity in seedling extracts of grass pea and pea.

Seedlings	vDAO Activity (U/mL)	Total Volume (mL)	vDAO Total Activity (**U)	Protein Concentration (mg/mL)	vDAO Specific Activity (U/mg Protein)
*Lathyrus sativus*	2.21 ± 0.09	45	99.45 ± 4.05	1.57 ± 0.47	1.41 ± 0.06
CDC Amarillo	1.31 ± 0.01	45	58.95 ± 0.45	1.71 ± 0.17	0.77 ± 0.01
CDC Meadow	0.82 ± 0.01	45	36.97 ± 0.61	1.30 ± 0.04	0.63 ± 0.01
CDC Limerick	0.75 ± 0.02	45	33.82 ± 0.99	1.84 ± 0.15	0.41 ± 0.02
CDC Inca	0.70 ± 0.01	45	31.52 ± 0.73	1.86 ± 0.21	0.38 ± 0.02
* Yellow pea	0.50 ± 0.03	45	22.50 ± 1.52	1.35 ± 0.09	0.36 ± 0.02
CDC Dakota	0.40 ± 0.01	45	18.40 ± 0.68	1.48 ± 0.08	0.27 ± 0.01

* Commercial yellow pea (unknown cultivar). **U one unit (U) is the amount of DAO needed to catalyse the oxidation of 1 µmol of putrescine/min. The values were obtained starting from 20 g of frozen seedlings. Data represent the results of three replicates (*n* = 3) and are expressed as mean ± SDs.

### 2.2. vDAO Activity during Germination of CDC Amarillo Seedlings

CDC Amarillo was selected to continue the investigation on DAO quantitation instead of *L. sativus* (grass pea), because of the known presence of β-ODAP in grass pea [31,32]. The DAO enzyme activity and specific activity in seedlings of CDC Amarillo increased over the course of 8 germination days, going from 0.44 U/mL and 0.19 U/mg on the 4th day to 1.08 U/mL and 0.81 U/mg on the 8th day. Beyond the 8th day, the vDAO activity was seen to decrease until it reached 0.66 U/mL and 0.47 U/mg on the 10^th^ day of germination (Table 2).

### 2.3. vDAO Activity in Seedling Organs

The distribution of vDAO activity in different organs of *CDC Amarillo* seedlings is summarized in Table 3. The vDAO specific activity in the shoots was 10- and 5-fold greater than in the cotyledons and roots, respectively, in 5-day-old seedlings. As mentioned, the enzymatic activity of vDAO in *L. sativus* extracts was the highest among all varieties analysed. However, due to the potential problems related to the presence of the neurotoxin β-ODAP in *L. sativus*, we developed a method that can be used to quantify β-ODAP with high specificity and sensitivity in seedling extracts.

**Table 2:** Effect of germination time on DAO activity in CDC Amarillo seedlings.

DAYS	vDAO Activity (U/mL)	Total Volume (mL)	vDAO Total Activity (U)	Protein Concentration (mg/mL)	vDAO Specific Activity (U/mg Protein)
1	* NA	NA	NA	NA	NA
2	NA	NA	NA	NA	NA
3	NA	NA	NA	NA	NA
4	0.44 ± 0.02	4.1	1.80 ± 0.08	2.37 ± 0.18	0.19 ± 0.01
5	0.59 ± 0.05	7	4.13 ± 0.35	2.74 ± 0.18	0.22 ± 0.02
6	0.68 ± 0.08	7	4.76 ± 0.56	1.29 ± 0.11	0.53 ± 0.06
7	0.84 ± 0.11	20	16.82 ± 2.2	1.39 ± 0.01	0.61 ± 0.07
8	1.08 ± 0.06	29	31.32 ± 1.74	1.34 ± 0.25	0.81 ± 0.18
9	0.91 ± 0.02	32	29.12 ± 0.64	1.52 ± 0.16	0.59 ± 0.04
10	0.66 ± 0.04	17	11.22 ± 0.68	1.41 ± 0.01	0.47 ± 0.02

* NA means non-applicable because the seedlings were only harvested after the fourth day of germination (no shoots were available for extraction in the first three days). Data represent the results of three replicates (*n* = 3) and are expressed as mean ± SDs. The volumes are different because the quantity of seedlings harvested was not the same each day of extraction.

**Table 3:** vDAO activity in different organs of the pea variety CDC Amarillo after 5 days of germination.

CDC Amarillo	vDAO Activity (U/mL)	Volume (mL)	vDAO Total Activity (U)	Protein Concentration (mg/mL)	vDAO Specific Activity (U/mg Protein)
Cotyledons	0.44 ± 0.04	5	2.22 ± 0.24	13.54 ± 0.38	0.03 ± 0.01
Roots	0.12 ± 0.006	5	0.59 ± 0.01	1.78 ± 0.23	0.06 ± 0.01
Shoots	0.84 ± 0.05	5	4.21 ± 0.22	2.74 ± 0.09	0.30 ± 0.03

Data from three replicates (*n* = 3) are expressed as mean ± SDs. The germination time was limited to 5 days because after this period the cotyledon reserves were nearly depleted.

### 2.4. LC-MRM Method Development and β-ODAP in Seedling Extracts

LC-MRM was used for the detection and quantitation of β-ODAP without the need for sample derivatization. Glycyl-L-aspartic acid (GlyAsp) was chosen as an appropriate internal standard (IS) to correct for losses that occur during sample preparation and analysis, for more precise quantitation. This IS was chosen because of its physicochemical properties, which were similar to those of β-ODAP.

An Acclaim Organic Acid LC column (150 × 2.1 mm, 3 µm) was used for the separation of β-ODAP, because of its ability to retain organic acids and the fact that the IS co-eluted under the conditions chosen, for better correction of matrix effects. Three MRM transitions were chosen for the detection of β-ODAP to ensure specificity of detection in real samples. A calibration curve between 0.5 and 50 µM was employed to quantify β-ODAP in extracts, which were diluted appropriately to fall into the dynamic range required by the method.

**Figure 1:**
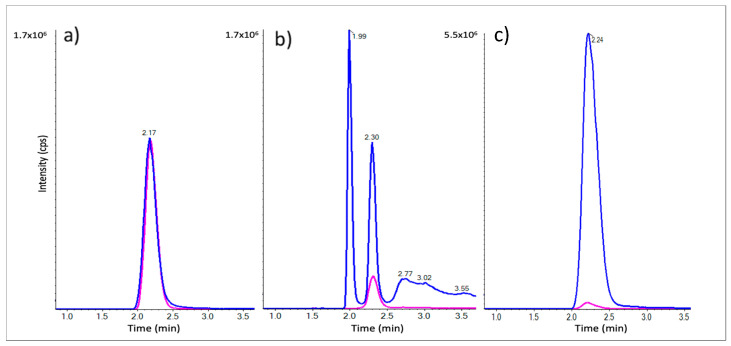
LC-MRM chromatograms of β-ODAP in blue (*m*/*z 177.0 → 116.0*) and Gly-Asp in pink (*m*/*z 191.0 → 116.0*) for a standard mix of 12.5 µM each (**a**) and *L. sativus* raw extract spiked with Gly-Asp following protein precipitation with ACN (**b**) and with the MAX-SPE step added (**c**).

The LC-MRM method was developed to detect analytes and IS with a good peak shape, as seen for the standard mix in Figure 1a. The analysis of raw *L. sativus* extracts following a simple protein precipitation step yielded unsatisfactory results, with the presence of multiple peaks and considerable peak tailing (Figure 1b). The irregular shape of the β-ODAP peak was likely due to the presence of remaining high salt concentrations. To facilitate detection and improve the peak shape of β-ODAP, a mixed-anion exchange (MAX) solid-phase extraction (SPE) step was added following protein precipitation. This protocol yielded an improved peak shape matching that of the standard solution, with perfect co-elution with the IS signal and improved sensitivity (Figure 1c).

### 2.5. β-ODAP Quantitation in DAO Extracts

**Figure 2:**
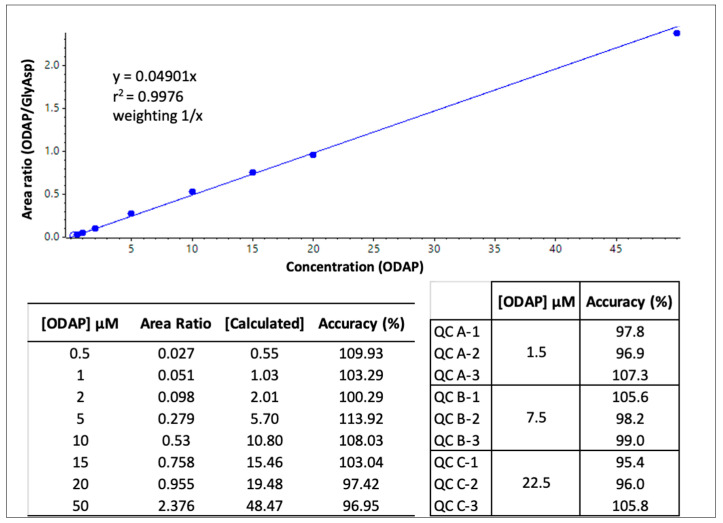
β-ODAP calibration curve, using the area ratio of ODAP/Gly-Asp LC-MRM peaks, as well as accuracy data for each calibration standard and triplicate QC samples at three concentration levels.

The quantitation of β-ODAP in crude extracts of *L. sativus,* CDC Amarillo, and yellow pea of an unknown cultivar (purchased from the supermarket) was assessed. The method was validated using an external calibration curve from 0.5 to 50 µmol/L β-ODAP in 0.1 mg/mL BSA, with the same sample preparation procedure as that used for the samples. Quality control samples (QCs) at three concentration levels were also prepared and extracted in the same manner. Figure 2 shows the calibration curve and accuracy data for the standards and QCs analysed with the sample extracts, which were all within the acceptance criteria of a validated quantitative assay.

**Figure 3:**
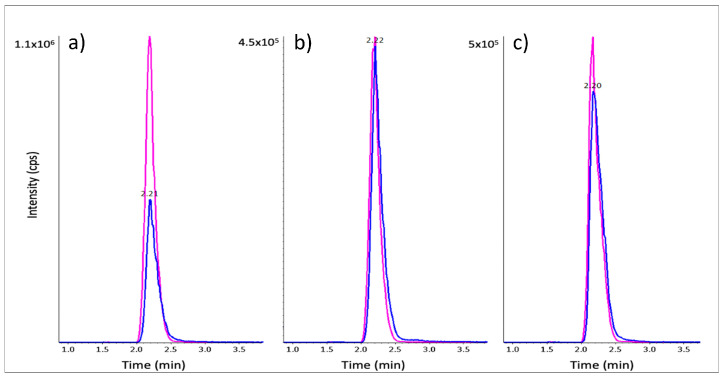
Representative LC-MRM chromatograms of β-ODAP (blue) and of Gly-Asp (pink) in a calibration standard at 10 µM β-ODAP in 0.1 mg/mL BSA (**a**), *L. sativus* crude extract (diluted 200-fold) (**b**) and dialysed extracts (diluted 10-fold) (**c**).

Figure 3 shows representative chromatograms of a calibration standard at 10 µM β-ODAP, as well as *L. sativus* crude extract before and after dialysis. The β-ODAP peak was significantly reduced after dialysis, considering the sample dilution (200-fold) for the raw extract prior to extraction to ensure the sample would fall into the dynamic range of the calibration curve. In Table 4, the β-ODAP concentration measured in DAO extracts is shown. *L. sativus* raw extract had on average 7.65 µmol ODAP/mg protein, and dialysis was shown to reduce this level substantially to 0.18 µmol ODAP/mg protein, whereas the β-ODAP concentrations measured in crude CDC Amarillo and in yellow pea extracts were approximately 5000 times lower (Table 4).

## 3. Discussion

The objective of this research was to evaluate the enzyme activity of vDAO during the germination of seeds from different legumes, i.e., *Lathyrus sativus* (grass pea) and *Pisum sativum* (pea). A selective and sensitive method was also developed to measure the concentration of the neurotoxin β-N-Oxalyl-L-α,β-diaminopropionic acid (β-ODAP) in the crude extracts obtained from their sprouts. In the evaluation of vDAO enzymatic activity (Table 1) from seedling extracts, it was revealed that vDAO is most active in *L. sativus*, followed by the pea cultivar CDC Amarillo.

During the first 10 days of germination of CDC Amarillo seedlings (Table 2), the vDAO enzyme activity and its specific activity were higher on the 8th day (1.08 U/mL and 0.81 U/mg protein) of germination, after which they decreased from the 9th day until they reached 0.66 U/mL and 0.47 U/mg protein on the 10th day of germination. These data are comparable with previous work by Luhova et al. [22] showing that *P. sativum* seedlings that germinated for 6 to 8 days contained more DAO than those that were allowed to grow for a longer period. In addition, the germination conditions in the dark may have led to an increase in vDAO activity over time. When plants undergo stress, such as that induced by growing in etiolated conditions, the amount of polyamine rises, leading to an increase in vDAO activity [33]. During germination, vDAO activity was higher in lentils (*Lens culinaris Medicus*) and peas (*Pisum sativum*) in etiolated seedlings than in light-grown ones [34].

In CDC Amarillo, vDAO enzyme activity was measured in different organs (roots, seeds, and shoots) of 5-day-old seedlings (Table 3). Both vDAO enzyme activity and specific activity were higher in shoots than in other organs. More precisely, the vDAO specific activity was found to be 10 and 5 times higher in shoots than in cotyledons and roots, respectively, whereas it was undetectable in seeds during the first 3 days of germination. Similar results were found in germinated fava beans (*Vicia faba* L.), where the DAO activity in shoots was significantly higher than that in seeds, radicles, cotyledons, and hypocotyls [35].

Another equally important objective of this study was to ensure that the chosen source of vDAO is safe and without health risk. There are several analytical methods in the literature (including colorimetric methods, gas chromatography–mass spectrometry, capillary zone electrophoresis, and flow injection analysis) for the detection of β-ODAP in samples of *L. sativus*. However, they can be time-consuming, with some requiring derivatization of the sample and being less effective for quantitation [36]. Liquid chromatography–tandem mass spectrometry (LC-MS/MS) is the most suitable analytical method, being the most selective and reliable [37]. For better sensitivity and selectivity of β-ODAP detection, sample preparation was optimized using raw extracts. A protein precipitation step followed by solid-phase extraction reduced matrix effects and improved peak shape, allowing much better detection [38] through the removal of excess salts and proteins present in crude extracts. The results (Figure 1b) show poor peak resolution with the presence of peak tailing as well as signal suppression. However, Figure 1c underlines the effectiveness of the precipitation of acetonitrile coupled with SPE clean-up to improve the resolution peak shape and signal of β-ODAP.

Vegetal DAO has been proposed for oral administration via monolithic tablets to ensure preservation of enzymatic activity in simulated gastric fluids [39]. Based on our results, the vDAO enzymatic activity in *L. sativus* was the highest among all varieties of legumes analysed. Because a previous study in the literature has mentioned the possible presence of β-ODAP in *L. sativus*, which is implicated in neurolathyrism [40], our objective was to ensure the utilization of a source of vDAO that is considered as GRAS (*generally recognized as safe*) and not subject to pharmacovigilance limitations.

The results of β-ODAP quantitation (Figure 2 and Table 4) show that it is present in a relatively high concentration in the crude extract of *L. sativus*, while it is 5000-fold less concentrated in the crude extract of CDC Amarillo. Since vDAO was suggested to be administered as monolithic tablets, a sample of freeze-dried *L. sativus* was also analysed with undetectable levels. The freeze-drying process (at low pressure [41,42]) and dialysis involved in the preparation of the powdered sample reduced the quantity of β-ODAP.

The toxicity level of β-ODAP detected in the crude extract of *L. sativus* was estimated according to data in the literature. In one study, neonatal and young mice that were injected intraperitoneally with high doses of β-ODAP (225–1350 mg/kg body) or via gavage (3900 mg/kg) developed seizures and opisthotonus [43]. Other in vivo studies showed mice exhibited signs of neurolathyrism 2–4 weeks after being given 300 mg/kg body/day of synthetic β-ODAP [44]. A level of ODAP in a legume cultivar below 0.2% (*w*/*w* seeds) is considered to be safe for human consumption [45]. Based on these data, theoretically, the threshold of β-ODAP considered safe for consumption is 0.3 g of β-ODAP/kg body/day. Since vDAO tablets are intended for humans and considering the average human adult weight is 80 kg, the safety threshold for β-ODAP is estimated at 24 g/day for 2–4 weeks. Considering the content of β-ODAP in *L. sativus* of 0.5–2.5% [14], the estimated toxic amount of *L. sativus* would be 960 g/day. Based on a 500 mg tablet containing vDAO at 20–60% weight, β-ODAP should not be present in quantities over 2.5–7.5 mg/tablet. Supposing an administration of 3 tablets/day, the estimated amount of ODAP may be 7.5 to 22.5 mg/day, which is much lower than the daily limit of toxicity.

Practically, a volume of 15 mL of extract of vDAO generates 1 g of powder after freeze drying. The mass of β-ODAP equivalent to the concentration of 4722 µmol/L before the dialysis is about 0.011 g. Even without dialysis and considering a consumption of 3 tablets/day, this amount is much lower than the 24 g/day of β-ODAP (or 960 g/day of *L. sativus*), which is suggested to be toxic for humans. In contrast to *L. sativus*, the two other pea varieties tested were essentially free of β-ODAP, providing a safe and convenient source of vDAO.

## 4. Materials and Methods

### 4.1. Materials

*Lathyrus sativus* seeds (chickling vetch, variety unknown) were kindly provided by Cover Crop Canada. *Pisum sativum* seeds: CDC Amarillo, CDC Meadow, CDC Limerick, CDC Inca, and CDC Dakota were generously provided by CDC (Crop Development Centre), University of Saskatchewan, commercial yellow pea (unknown cultivar), bovine serum albumin (BSA), horseradish peroxidase (HRP, Type I, 96 U/mg solid), putrescine (1,4-diaminobutane dihydrochloride), 4-amino-antipyrine (AAP), 3,5-dichloro-2-hydroxybenzenesulfonate sodium (DCHBS), β-N-oxalyl-L-α,β-diaminopropionic acid (β-ODAP), and glycyl-L-aspartic acid (Gly-Asp). Membranes of cellulose (12–14 kDa cut-off) were purchased from Sigma–Aldrich (St. Louis, MO, USA). Bradford reagent and the acrylamide/bis-acrylamide solution (29:1) were purchased from Bio-Rad Laboratory (Mississauga, ON, Canada). All other chemicals were reagent-grade and were used without further purification.

### 4.2. Germination of Seeds

The pea and grass pea seeds were sterilized using 10% (*v*/*v*) of sodium hypochlorite for 20 min, and then washed and imbibed in distilled water at room temperature in the dark for 16–20 h. The soaked seeds were placed in a germination chamber and germination was carried out in trays (54 cm length × 27 cm width containing two bins 24 cm length × 24 cm width) cleaned and disinfected beforehand with bleach and then covered with a plastic cover with holes. The germination conditions were as follows: temperature 25 °C, relative humidity 80%, in the dark. A culture solution or running water was given every 12 h for seed germination until seedlings about 9 to 12 cm in length were obtained. After 8 days of germination, the seedlings were collected and washed with distilled water, and then stored at −80 °C prior to measuring vDAO activity, protein, and β-ODAP content.

### 4.3. vDAO Extraction

Frozen seedlings were ground with a blender until obtention of small uniform pieces (a few millimetres in size) and then mixed under stirring at a ratio of 1:2 (mass/volume) with 50 mM sodium phosphate buffer containing 200 mM of NaCl pH 5.5. After 30 min, the mix was filtered and then centrifuged at 10,000 rpm for 10 min at 4 °C. The supernatants were stored at −20 °C for further analyses.

### 4.4. Spectrometric Assay of vDAO Activity

Measurement of H_2_O_2_ formation was conducted using the DCHBS AAP−HRP method using putrescine as a substrate [46]. The vDAO activity was determined using reaction solutions (1.6 mL) containing 1 mL of sodium phosphate buffer, 0.1 M (pH 7.4), 30 µL of putrescine, 100 mM, 10 µL of 4-aminoantipyrine, 100 mM, 10 µL of 3,5-Dichloro-2-hydroxybenzenesulfonic acid, 100 mM, 10 µL of horseradish peroxidase, 1 mg/mL. The reaction was initiated by adding 50–100 µL of crude enzyme extract. The absorbance at 515 nm was read on a PerkinElmer LS45 fluorimeter (PerkinElmer, Waltham, MA, USA). vDAO activity was measured in 8-day-old seedlings.

### 4.5. Dialysis of Raw Extracts

The supernatant obtained after the vDAO extraction was dialysed overnight using cellulose membranes (12–14 kDa cut-off) with constant mixing in a beaker with distilled water. Prior to dialysis, the membranes were treated with 0.3% (*w*/*v*) of sodium sulphide at 80 °C for 1 min, and then washed with hot water (60 °C) for 2 min, followed by acidification with 0.2% (*v*/*v*) sulfuric acid, with a final rinse with hot water. The distilled water was changed 3 times over a 24 h period.

### 4.6. ODAP Calibration Curve Preparation and Validation of Quantitative Analysis

Standard stock solutions of β-ODAP and Gly-Asp at 10 mM concentrations were prepared in 10% ACN and 10% ACN with 0.1% ammonium hydroxide, respectively. All subsequent standard dilutions were prepared in 10% ACN. A standard calibration curve was prepared using 0, 0.5, 1, 2, 5, 10, 15, 20, and 50 µM concentrations of β-ODAP in 0.1% BSA. Quality control (QC) samples were prepared in triplicate at three concentration levels, 1.5, 7.5, and 22.5 µM, also in 0.1% BSA. The final linear calibration curve was weighted at 1/x, passing through zero. Acceptance criteria for standards and QC samples were within accuracies of 85–115%.

### 4.7. Sample Preparation for β-ODAP Analysis

Raw *L. sativus* and *P. sativum* DAO extracts were initially tested with a simple acetonitrile precipitation step for sample preparation for ODAP quantitation. Briefly, 250 µL of extract was combined with internal standard Gly-Asp (at 25 µM) and 3 volumes of ice-cold acetonitrile. Samples were put at −30 °C for 10 min, and then centrifuged at 14,000 rpm for 10 min at 4 °C. Supernatants (750 µL) were transferred to new tubes and dried in a SpeedVac universal vacuum system (ThermoFisher Scientific, Asheville, NC, USA). Samples were reconstituted in 100 µL of 10% acetonitrile.

The optimized sample preparation method combining protein precipitation and solid-phase extraction was used for the analysis of raw extracts from *Lathyrus sativus,* CDC Amarillo, *Pisum sativum* (yellow pea, unknown cultivar), and dialysed *Lathyrus sativus,* provided in triplicate samples. Each sample was subjected to protein quantitation using the Bradford method [47], using BSA as the calibration standard, for normalization of ODAP content. Some samples were then diluted to fall within the dynamic range of the calibration curve. For all samples, standards and QCs, a volume of 200 µL was mixed with 50 µL of 100 µM Gly-Asp internal standard solution, followed by the addition of 3 volumes of ice-cold ACN as described above. After drying the supernatants, they were reconstituted in 1 mL of 0.1% ammonium hydroxide in water. Solid-phase extraction was performed using Oasis MAX 1 cc/30 mg cartridges (Waters Limited, Mississauga, ON, Canada). Each cartridge was conditioned with methanol, and then water, before loading the samples. Cartridges were washed with 1 mL of 0.1% ammonium hydroxide and 500 µL of water. Samples were then eluted with 5% formic acid in methanol in two steps of 500 µL. Eluates were dried then reconstituted in 200 µL of 10% ACN for analysis. Samples were analysed using LC-MRM in a randomized order.

### 4.8. LC-MRM Analysis

Final extracted samples, standards, and QCs (5 µL) were analysed on a QTRAP 5500 mass spectrometer (Sciex, Concord, ON, Canada) coupled to a Nexera UHPLC (Shimadzu, Columbia, MD, USA) equipped with a Thermo Acclaim Organic Acid C18 column (2.1 × 150 mm, 3 µm). Mobile phases were water (A) and acetonitrile (B), both containing 0.1% formic acid, at 40 °C at a flow rate of 0.2 mL/min. The elution gradient was as follows: 3% B held for 1 min, and then increased linearly to 25% B at 5 min, up to 90% B at 5.5 min. MRM experiments were performed in positive electrospray ionization (ESI) mode with the following source parameters: ion spray voltage, 5000 V; temperature, 500 °C; nebulizer and drying gases (GS1 and GS2), 50 psi; and curtain gas, 35 psi; declustering potential, 80 V. Quantitation was performed using the MRM transitions *m*/*z 177.0* → *116.0* (analyte β-ODAP) and *m*/*z 191.0* → *116.0* (IS Gly-Asp) with collision energies of 13 and 25 V, respectively, and dwell times of 150 ms. Other qualitative transitions were followed for both compounds at *m*/*z 177.0* → *88.0* and *59.0* for β-ODAP, and *m*/*z 191.0* → *134.0* and *74.0* for Gly-Asp. Quantitative analysis was carried out using Sciex OS-Q v2.0.1.

## 5. Conclusions

This study demonstrated that *Pisum sativum* cultivars are an interesting source for vDAO extraction. Of the five *P. sativum* cultivars evaluated, CDC Amarillo had the greatest vDAO concentration. It is important to note that CDC Amarillo was developed at the University of Saskatchewan, and has a strong agronomic performance, good fungal disease resistance, high grain yield, high seed protein concentration, and is widely grown in western Canada [48,49,50,51]. CDC Amarillo is also widely available for vDAO extraction. *L. sativus* (grass pea) was the source containing the most active vDAO tested. A simple and reliable procedure was described to evaluate the concentration of the neurotoxin β-ODAP in crude extracts. Since both genotype and environmental conditions [52] can modify the content of β-ODAP in grass pea seeds, it is important to have a convenient method such as LC-MS to detect this compound. Overall, it was shown that seedlings of *L. sativus* and *P. sativum* can be used as vegetal sources of vDAO that are safe for humans when administrated in oral formulations to treat food histaminosis and to prevent histamine-related dysfunctions.

## Figures and Tables

**Table 4:** β-ODAP concentrations found in *L. sativus,* CDC Amarillo, and yellow pea extracts. An external calibration curve of β-ODAP was used for quantitation, using Gly-Asp as the internal standard.

Samples	Protein Content (µg/mL)	[β-ODAP] (µmol/L)	[β-ODAP] (µmol/mg Protein)
*L. sativus* crude extract	600 ± 120	4722 ± 2116	7.65 ± 2.02
*L. sativus* dialysed extract	876 ± 219	158 ± 35	0.18 ± 0.02
CDC Amarillo crude extract	624 ± 115	0.99 ± 0.03	0.0016 ± 0.0003
* Yellow pea crude extract	615 ± 181	0.58 ± 0.16	0.0011 ± 0.0006

* Commercial yellow pea (unknown cultivar). Data represent results of three replicates (*n* = 3) and are expressed as mean ± SD.

## Abbreviations

4-Aminoantipyrine (AAP); acetonitrile (ACN); bovine serum albumin (BSA); β-N-Oxalyl-L-α,β-diaminopropionic acid (β-ODAP); Crop Development Centre (CDC); diamine oxidase (DAO); 3,5-Dichloro-2-hydroxybenzoic acid (DCHBS); electrospray ionization (ESI); formic acid (FA); Glycyl-Aspartate (Gly-Asp); histamine intolerance (HIT); horseradish peroxidase (HRP); internal standard (IS); mixed-mode anion exchange (MAX); *Lathyrus sativus* (*L. sativus*); liquid chromatography–mass spectrometry (LC/MS); multiple-reaction monitoring (MRM); *Pisum sativum* (*P*. *sativum*); quality control samples (QCs); solid-phase extraction (SPE); vegetal diamine oxidase (vDAO).
